# Polycomb repressive complex 2 controls cardiac cell fate decision *via* interacting with RNA: Promiscuously or well-ordered

**DOI:** 10.3389/fgene.2022.1011228

**Published:** 2022-10-14

**Authors:** Gang Wang, Heng Ye, Xuchao Wang, Binbin Liu

**Affiliations:** ^1^ Xiamen Cardiovascular Hospital, School of Medicine, Xiamen University, Xiamen, China; ^2^ School of Pharmaceutical Engineering, Shenyang Pharmaceutical University, Shenyang, Liaoning, China; ^3^ School of Pharmacy, Shenyang Pharmaceutical University, Shenyang, Liaoning, China

**Keywords:** heart development, PRC2, promiscuous RNA binding, lncRNA, cardiac cell fate decisions

## Abstract

The epigenetic landscape determines cell fate during heart development. Polycomb repressive complex 2 (PRC2) mediates histone methyltransferase activity during cardiac cell differentiation. The PRC2 complex contains the proteins embryonic ectoderm development (EED), suppressor of zeste (SUZ12), the chromatin assembly factor 1 (CAF1) histone-binding proteins RBBP4 and RBBP7, and the histone methyltransferase called enhancer of zeste (EZH2 or EZH1), which incorporates the Su(var)3-9, Enhancer-of-zeste, Trithorax (SET) domain. Cardiac PRC2-deficient mice display lethal congenital heart malformations. The dynamic process of cardiac cell fate decisions is controlled by PRC2 and the PRC2-mediated epigenetic landscape. Although specific individual long noncoding RNAs (lncRNAs) including *Braveheart* were widely reported to regulate the recruitments of PRC2 to their specific targets, a promiscuous RNA binding profile by PRC2 was also identified to play an essential role in cardiac cell fate decision. In this review, we focus on RNA-mediated PRC2 recruitment machinery in the process of cardiac cell fate decisions. The roles of individual lncRNAs which recruit PRC2, as well as promiscuous RNA binding by PRC2 in heart development are summarized. Since the binding priority of RNAs with different primary and secondary structures differs in its affinity to PRC2, the competitive relationship between individual lncRNAs binding and promiscuous RNA binding by PRC2 may be important for understanding the machinery by which biding of individual lncRNA and promiscuous RNA by PRC2 coordinately control the well-ordered dynamic cardiac cell lineage differentiation process.

## 1 Introduction

**FIGURE 1:**
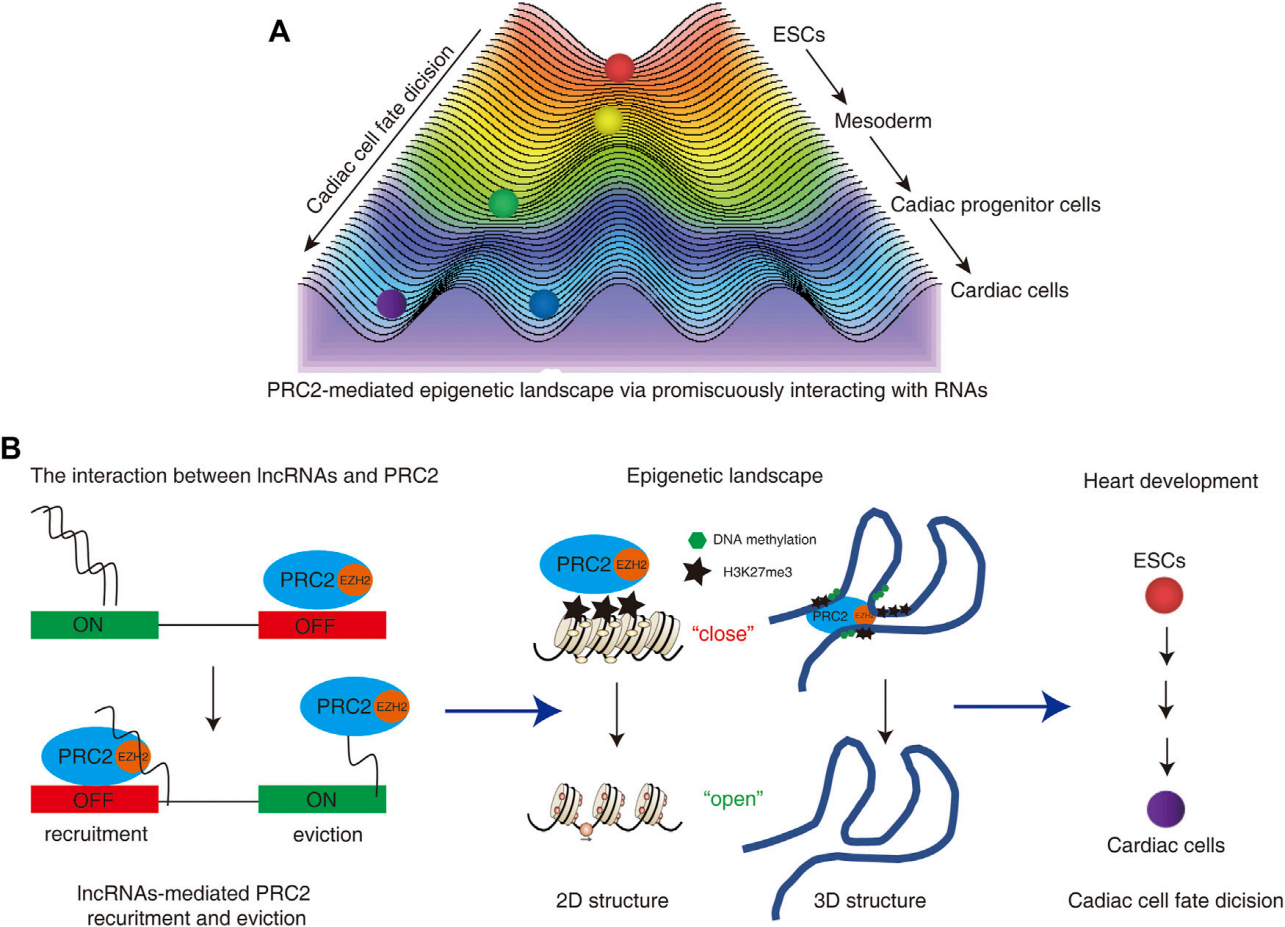
**(A)** PRC2-mediated epigenetic landscape controls cardiac cell fate decision. Stem cells are reimagined as pebbles on the top of a hill. During development, stem cells differentiate into different types of cells as the pebbles roll down from the top. The final differentiated cells are the cell fates of these cells. Epigenetic-paved pathways like small paths on a hill decide the destination of these cells. **(B)** The interactions between lncRNAs and PRC2 regulate the functions and occupies of PRC2 on the genomes. The dual roles of PRC2-RNA interaction regulate the recruitments and/or eviction of PRC2 from the genome, which further “turn on” or “turn off” the gene expressions. The distribution of PRC2 could induce the formation of heterochromatin or heterochromatin-like structures *via* deposition of repressed histone modification marker H3K27me3 on the 2D and 3D genome. The dynamic changes of PRC2-mediated epigenetic landscape decide the cardiac cell fate and regulate heart development.

Both genetics and epigenetics are focused on the study of genes and heredity. Genetics studies on how certain qualities or traits are passed on from parents to offspring as a result of changes in DNA sequence, while epigenetics is focused on the study of heritable phenotypic changes that do not involve alterations in DNA sequence (Dupont et al., 2009). Epigenetic modifications include DNA methylation and histone modifications, which regulate gene expression by altering DNA accessibility and 3-dimensional (3D) chromatin organization. During development, dynamic changes in the epigenetic profile control the transcriptional program which decides cell fate and function (Atlasi and Stunnenberg, 2017). The epigenetic landscape, as proposed by Conrad Waddington in 1957, is an abstract metaphor that is frequently used to represent the relationship between gene activity controlled by epigenetic profile and cell fates during development (Allen, 2015). Stem cells are reimagined as pebbles on the top of a hill. During development, stem cells differentiate into different types of cells as the pebbles roll down from the top (Moris et al., 2016). The final differentiated cells are the cell fates of these cells. Epigenetic-paved pathways like small paths on a hill decide the destination of these cells (Figure 1A). Thus epigenetic landscapes decide cell fate during development.

The heart is the first functional organ emerging from the mesoderm during embryogenesis (Christoffels and Jensen, 2020). The process of heart development involves a series of cell fate decisions and morphological changes. The well-studied first heart field (FHF) is differentiated from anterior lateral plate mesodermal cells at week 2 of human gestation. The first cardiac cell lineage differentiation occurs at FHF, while the second heart field (SHF) contains ISL LIM Homeobox 1 (ISL1)^+^ cardiovascular progenitors which give rise to the cardiomyocyte, smooth muscle and endothelial cell lineages at a relatively delayed time point (Srivastava, 2006; Bu et al., 2009). Both cell lineages are regulated by a complex network including multiple transcription factors, growth factors and cell membrane proteins (Zaffran and Frasch, 2002). The finely tuned gene expression network is regulated by a dynamic epigenetic landscape which controls the conformation of chromosome and the access of transcription factors to consensus DNA (George and Firulli, 2021).

The polycomb group (PcG) proteins, originally found in *Drosophila melanogaster*, are epigenetic regulators that maintain the transcriptional silence of numerous genes, most of which encode developmental and/or cell cycle regulators (Oktaba et al., 2008). In mammals, PcG machinery has been subdivided into polycomb repressive complex 1 (PRC1) and 2 (PRC2), which contribute to transcriptional repression of many genes and affect development and pluripotential embryonic stem cells (ESCs) maintenance during early embryonic development. (Schuettengruber et al., 2017). The functional core of the PRC2 complex includes the proteins embryonic ectoderm development (EED), suppressor of zeste (SUZ12), the chromatin assembly factor 1 (CAF1) histone-binding proteins RBBP4 and RBBP7, and the histone methyltransferase called enhancer of zeste (EZH2 or EZH1), which incorporates the Su(var)3-9, Enhancer-of-zeste, Trithorax (SET) domain. PRC2 mediates histone methyltransferase activity (Blackledge et al., 2015). The catalytic SET-domain of EZH1/2 methylates histone H3 on lysine residue 27 (H3K27me) to produce H3K27me2/3, which further interacts with EED to stimulate the successive methyltransferase activity of PRC2 and induces facultative heterochromatin formation (Cao et al., 2002; Jiao and Liu, 2015, 1).

In this review, we summarize the role of the PRC2 complex in heart development and cardiac cell fate decisions, focusing on the roles of RNA in the PRC2-mediated epigenetic landscape during cardiac cell fate decision-making. An updated promiscuous PRC2 recruitment model is presented which reconsiders the relationship between specific individual RNA binding and promiscuous RNA binding by PRC2, and how both coordinately controls the well-ordered process of dynamic cardiac cell lineage differentiation.

## 2 PRC2 functions in heart development

### 2.1 PRC2 regulates normal development of the heart

PRC2 has been detected in the zygote and contributes to facultative heterochromatin establishment across the zygote genome (Meng et al., 2020). During early development, EZH2 is broadly expressed throughout the gastrulating and post-gastrulating mouse embryo. Null mutations of the PRC2 subunits, EZH2 and SUZ12, result in lethality at early stages of mouse development (O’Carroll et al., 2001; Pasini et al., 2004), which suggests that PRC2 plays a key role in early embryonic development. Inactivation of EZH2 in a cardiac cell lineage caused lethal congenital heart malformations, namely compact myocardial hypoplasia, hypertrabeculation and ventricular septal defect (He et al., 2012). Moreover, the Jumonji- and AT-rich interaction domain (ARID)-domain-containing protein (JARID2) could form a stable complex with the core PRC2 complex and regulate binding of PRC2 to targets (Takeuchi et al., 1999; Pasini et al., 2010). The deletion of *Jarid2* by cardiac cell lineage specific *NK2 homeobox 5* (*Nkx2.5*)*-Cre* mice induced similar cardiac malformations as EZH2-null mice including ventricular septal defects, thin myocardium, hypertrabeculation, and neonatal lethality (Cho et al., 2018). In addition, EZH2 and EZH1 double-deficient mice also expressed similar cardiac malformations (Ai et al., 2017b). Thus, these mice studies indicate that PRC2 is essential for normal heart development.

At 12.5 days post coitum (E12.5), cardiac EZH2 deficiency significantly changed the expression of 511 genes in the ventricle apex including *cyclin dependent kinase inhibitor 2A* (*Cdkn2a*), *SIX homeobox 1 (Six1)*, *Isl1*, *paired box 6 (Pax6)*, *myosin heavy chain 6 (Myh6) and hyperpolarization activated cyclic nucleotide gated potassium channel 4 (Hcn4)*, which are related to heart development (He et al., 2012). The direct binding sites of JARID2 control the expression of heart development-associated genes (Takeuchi et al., 1999). De-repression of *Isl1*, a maker gene of cardiovascular progenitors, was observed at E10.5 in JARID2-null mice indicating that H3K27me3-mediated gene silencing is essential for heart development (Takeuchi et al., 1999). Endothelial JARID2 was also reported to repress Notch1 expression in the endocardium and induce expression of notch receptor 1 (Notch1) in adjacent myocardium (Mysliwiec et al., 2011). Thus, the PRC2-controlled gene expression network may temporally and spatially regulate the heart development process.

H3K27me3, a direct product of PRC2, plays a key role in regulating heart development (Zhang and Liu, 2015). Genome-wide changes of H3K27me3, which regulates the gene expression network during heart development, have been observed in PRC2-deficent cardiac cells (Takeuchi et al., 1999; Pasini et al., 2007; He et al., 2012). A histone H3-lysine 27 demethylase UTX deficiency also induces severe cardiac malformations and expression changes in cardiac-specific genes during early embryonic development. (Lee et al., 2012). Thus, the PRC2-mediated epigenetic landscape is essential for heart development by directly changing both H3K27me3 distributions on cardiac-specific genes. EED, one of the PRC2 subunits, was reported to induce abnormal heart development by changing H3K27ac levels at genome but not the levels of H3K27me3 (Ai et al., 2017a). PRC2 could cooperate with H3K9 methylation to maintain heterochromatin (Boros et al., 2014; Frapporti et al., 2019). Thus, PRC2 may directly and indirectly alter both H3K27me3 and other epigenetic modifications of the genome during heart development.

In summary, PRC2 controls normal heart development by temporally and spatially regulating the expression of genome-wide cardiac-specific genes in an epigenetic manner during heart development (Figure 1B).

### 2.2 PRC2 regulates cardiac cell fate decisions

The first lineage choice in embryonic development separates trophectoderm from the inner cell mass (Kumar et al., 2022), and naïve human ESCs deriving from the inner cell mass are used to study cell fate decisions during early embryonic development. Directed differentiation of human ESCs into cardiomyocytes provides a model for studying the molecular mechanisms of human cardiac cell fate decisions (Mummery et al., 2003; Mummery et al., 2012; Breckwoldt et al., 2017). There are five key developmental stages during cardiovascular-directed differentiation which involve pluripotent cells, mesodermal progenitors, specified tripotential cardiovascular progenitors, committed cardiovascular cell, and definitive cardiovascular cells. Chromatin states measured along the time course of differentiation indicate that temporal chromatin signatures including the H3K27me3-binding profile regulate stage-specific expression of functionally related genes (Paige et al., 2012) and contribute to developmental transitions in the cardiac cell lineage (Wamstad et al., 2012). Thus, the dynamic PRC2-mediated H3K27me3-binding profile plays a key role in cardiac cell fate decisions during heart development.

Deletion of EZH2 in cardiac progenitors results in abnormal cardiac cell differentiation (Delgado-Olguín et al., 2012). Human induced pluripotent stem cells (iPSCs) with EZH2 mutations repressed the generation of cardiomyocytes during *in vitro* ESC differentiation (Long et al., 2020). Deletion of PRC2 in progenitors or stem cells induced premature expression of cardiac genes, including key transcription factors ladybird homeobox 2 (LBX2), forkhead box F2 (FOXF2) and Six1, which are critical for forthcoming developmental stages (Delgado-Olguín et al., 2012; Long et al., 2020). Repression by H3K27me3 occupancy in genome is essential for silencing these genes during the early development, and abnormal H3K27me3 occupancies-induced premature expression of development-associated genes could alter the cell fate decision during early embryonic development (Zhang et al., 2022). Thus, PRC2 and PRC2-induced H3K27me3 may rein in premature activation of cardiac genes during the forthcoming cardiac cell differentiation process to control the cardiac cell fate decisions.

In summary, PRC2 mainly functions as a gene silencer to deposit H3K27me3 on cardiac genes to rein in their premature activation. The temporal and spatial distributions of PRC2 and H3K27me3 control the gene expression network of heart development. The alteration of PRC2 distribution is associated with the abnormal heart development and cardiac cell fate decisions (Figure 1B).

## 3 lncRNAs-mediated PRC2 recruitment is essential for heart development

PRC2-mediated epigenetic landscape is associated with direct or indirect PRC2-binding on chromosomes. Clarification of the PRC2 recruitment machinery is therefore critical for understanding the dynamic changes in the epigenetic landscape during cardiac development. Since the core PRC2 component lacks a DNA binding domain, core PRC2 itself can not directly bind to the genome. Instead, transcription factors (Chang and Bruneau, 2012) and long noncoding RNAs (lncRNAs) (Rizki and Boyer, 2015) have been reported to recruit PRC2 to the genome during heart development. The role of both individual lncRNAs and promiscuous lncRNAs-mediated PRC2 recruitment machinery in heart development were summarized (Figure 1B).

### 3.1 The role of individual lncRNAs in regulation of cardiac cell fate decision by PRC2

During the process of *in vitro* cardiac differentiation and embryonic development, hundreds of lncRNAs are expressed in a stage-specific manner (Wamstad et al., 2012; Zhu S. et al., 2014, Zhu J. G. et al., 2014). The stage-specific lncRNAs function *in cis* to regulate the expressions of their nearest genes and control the cardiac cell fate decision, since the function of their nearest genes are enriched in development, morphogenesis, and transcriptional processes (Wamstad et al., 2012). *Braveheart* (*Bvht*) was the first identified lncRNA, which is expressed in the early stages of embryonic heart development, contributing to the formation of beating cardiomyocytes (Klattenhoff et al., 2013). The interaction between *Bvht* and PRC2 was observed to control cardiac cell fate decisions *via* mesoderm posterior 1 (MesP1), the master regulator of a common multipotent cardiovascular progenitor (Klattenhoff et al., 2013). The roles of individual lncRNAs and individual lncRNA-mediated PRC2 recruitment during cardiovascular development and disease have been reviewed (Rizki and Boyer, 2015; García-Padilla et al., 2018). Briefly, the majority of these individual lncRNAs can bring PRC2 to specific sites to control the expression of a few key genes which are essential for cardiac cell fate decisions. The binding sites of PRC2 are therefore specific, limited and well-ordered. However, individual lncRNA-mediated PRC2 recruitment has been challenged, since PRC2 was also reported to interact with RNA in a promiscuous manner (Davidovich and Cech, 2015). Thus, the role of PRC2 recruitment mediated by individual lncRNAs to specific sites may only play a limited role in regulating the epigenetic landscape during cardiac cell fate decision-making.

### 3.2 The role of promiscuous lncRNAs in regulation of cardiac cell fate decision by PRC2

#### 3.2.1 Promiscuous RNA binding by PRC2

PRC2 was reported to bind to multiple non-relevant RNAs, including bacterial mRNAs *in vitro* (Davidovich et al., 2015). Studies using photoactivatable ribonucleoside-enhanced crosslinking and immunoprecipitation indicated that PRC2 could interact with a specific set of 774 nascent RNAs (Kaneko et al., 2013). Individual-nucleotide-resolution crosslinking and immunoprecipitation (iCLIP) experiments demonstrated that PRC2 could interact with nascent, unspliced pre-mRNA from essentially all active genes (Skalska et al., 2017; Beltran et al., 2019). Thus, the interaction between PRC2 and RNA is promiscuous. The promiscuous binding of PRC2 means that PRC2 can bind to many RNAs without the requirement for an obvious protein-binding motif and with affinities that are not enormously different; furthermore, this promiscuous binding is not the same as nonspecific binding (Davidovich and Cech, 2015).

**FIGURE 2:**
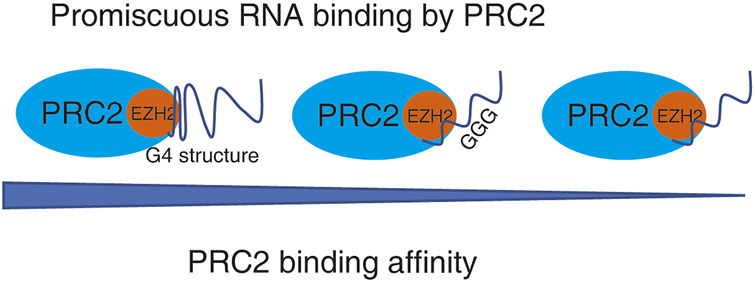
PRC2 binds to promiscuous RNAs. PRC2 binds to many RNAs without the requirement of an obvious protein-binding motif, while the binding affinity of PRC2 to RNAs with various structure is different. RNA with G4 structure has highest binding affinity to PRC2.

Moreover, iCLIP studies and *in vitro* binding experiments indicated that PRC2 has higher affinity to short repeats of consecutive guanines, and G-tract motifs are significantly enriched among PRC2-binding transcripts. Further, PRC2 has a high affinity to folded guanine quadruplex (G4) structures but shows little binding to duplex RNAs (Wang et al., 2017; Beltran et al., 2019). Although PRC2 can promiscuously interact with lncRNA and pre-mRNA, the affinity of PRC2 to individual RNAs still has some priority. The binding capacity of PRC2 to RNA is positively associated with the numbers of G4 structures in RNA (Figure 2). Thus, it is possible that individual RNAs with different structures may competitively interact with PRC2 and regulate PRC2 distributions in the genome.

#### 3.2.2 Dual roles of PRC2-RNA interaction in regulating gene expression

**FIGURE 3:**
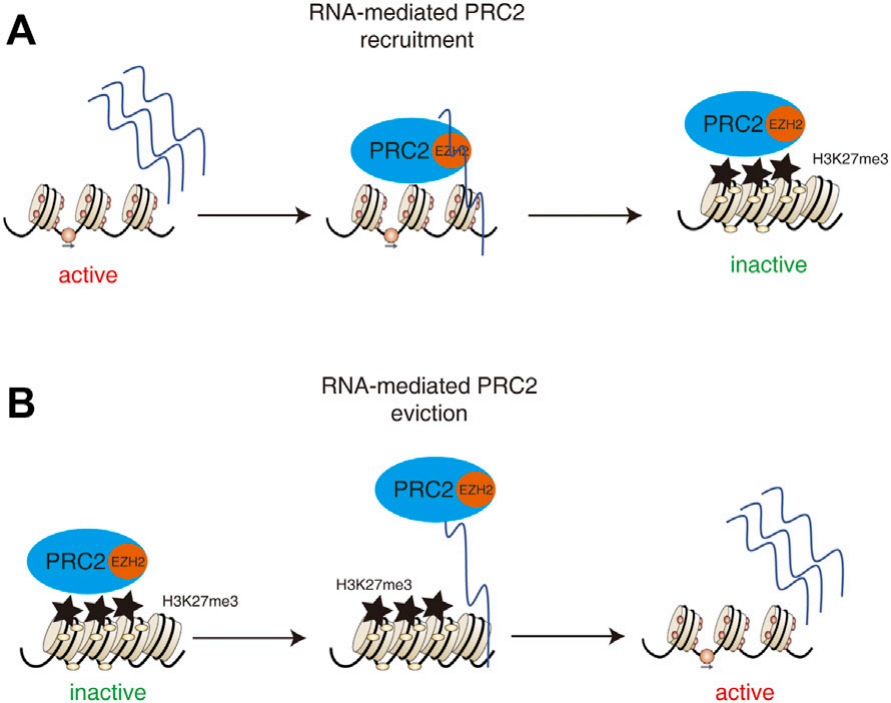
Dual roles of PRC2-RNA interactions in regulating gene expression. **(A)** The pre-mRNA and lncRNAs in activated expressed gene regions could recruit PRC2 and then deposits the repressor marker H3K27me3 onto the targets in order to repress the gene expression. Therefore, the transcriptionally activated regions can be silenced by PRC2 after the cell fate changed. **(B)** The interaction of PRC2 with RNA or chromatin is mutually antagonistic such that the interaction of PRC2 with pre-mRNA or lncRNAs can remove the binding of PRC2 from the genome and promote gene expression.

Whether direct PRC2-RNA interactions play any role in gene repression or activation remains an open question. PRC2 shows a tendency to scan for the actively transcribe genes by binding to pre-mRNA in activated expressed gene regions and then deposits the repressor marker H3K27me3 onto the targets in order to repress the gene expression. Therefore, the transcriptionally activated regions can be silenced by PRC2 after the cell fate changed (Long et al., 2017) (Figure 3A) On the other hand, the interaction of PRC2 with RNA or chromatin is mutually antagonistic (Beltran et al., 2016) such that the interaction of PRC2 with pre-mRNA can remove the binding of PRC2 from the genome and promote gene expression (Beltran et al., 2019). (Figure 3B) These controversial results suggest that the PRC2-RNA interaction plays a complex and context-dependent role in regulating gene expression during cell fate decisions.

For this reason, “junk mail model” has been proposed to explain the PRC2-induced gene expression phenotype. In this model, the PRC2 is the junk mail, chromosomal loci are mailboxes, transcriptional activity is the criterion for delivery, and the response (active or inactive) is dictated by the local chromatin context (Davidovich and Cech, 2015). However, what kinds of local chromatin context regulates or decides the response to PRC2 recruitment is still unknown.

#### 3.2.3 Promiscuous RNA binding by PRC2 controls cardiac cell fates

During the process of cardiac cell differentiation, iPSCs expressing the RNA-binding-defective mutant EZH2 could specially block the interaction between PRC2 and RNA without changing other functions of EZH2 such as complex assembly, DNA binding, nucleosome binding and methyltransferase activity (Long et al., 2020). Genome-wide EZH2 and H3K27me3 occupancies were lost in this mutant iPSCs. This suggests that global PRC2 occupancies are dependent on the interaction with promiscuous RNAs but not with specific RNA. Moreover, the mutant iPSCs could not generate the cardiac troponin T (cTnT)-positive cardiomyocyte during *in vitro* differentiation (Long et al., 2020). This suggests that promiscuous RNA binding by PRC2 is essential for cardiac cell fate decisions. However, it is still possible that some well-understood specific RNAs including *Bvht* may play key roles in regulating cardiac cell fate decisions, since the interactions with some specific RNAs would be lost in the mutant iPSCs.

In summary, promiscuous RNA binding by PRC2 is essential for global PRC2 occupancies and cardiac cell fate decisions. Furthermore, heart development requires a PRC2-dependent well-ordered and stage-specific epigenetic landscape. However, the underlying mechanisms by which promiscuous RNA binding by PRC2 dynamically controls the well-ordered and stage-specific epigenetic landscape during heart development is an unknown and important question.

## 4 Individual lncRNAs regulate a broader PRC2-dependent epigenetic structure during heart development

### 4.1 Individual lncRNAs regulate genome-wide PRC2 occupancy *via* microRNA

Since global PRC2 occupancies are essential for heart development, whether individual lncRNAs can regulate genome-wide gene expression and PRC2 binding is an interesting question. LncRNA heart brake lncRNA 1 (HBL1) was recently reported to control genome-wide PRC2 occupancies during the process by which human ESCs direct differentiation into cardiomyocytes (Liu et al., 2021). By counteracting the actions of miR-1 (Liu et al., 2017), HBL1 modulates the expression of transcription factor JARID2, which further controls the global PRC2 occupancy and cardiogenic gene transcription (Liu et al., 2021). Therefore, HBL1 deficiency-induced changes in genome-wide PRC2 occupancies are an indirect effect. Whereas HBL1 directly controls the epigenetic modifications of specific miR-1 gene loci. Thus, individual lncRNA may indirectly regulate genome-wide gene expression and PRC2 occupancy *via* microRNA.

### 4.2 Individual lncRNAs regulate higher orders of genome architecture

PRC2 regulates chromatin dynamics at multiple levels including nucleosomal scale, a 2D chromatin organization, and supra-nucleosomal and nuclear scale, the 3D chromatin organizations (Pachano et al., 2019). Kcnq-overlapping lncRNA 1 (*Kcnq1ot1*), one imprinted lncRNA, was reported to recruit PRC2 on target genome and induce the spread of PRC-dependent chromatin modifications over multi-megabase domains. The spread of polycomb is controlled by genome architecture and CpG island DNA (Schertzer et al., 2019). *Kcnq1ot1* and its target gene *Kcnq1* are important for the maintenance of proper heart conduction (Harmer et al., 2014, 1). Thus, individual lncRNA may regulate broader gene expression *via* alteration of PRC- dependent higher orders of genome architecture during heart development.

In summary, individual lncRNAs-induced alterations of genome-wide PRC2 occupancies and higher orders of genome architecture could control a broader or genome-wide gene expressions during heart development. The dynamic changes in global PRC2 occupancies during heart development might be controlled by some individual lncRNAs *via* modification of higher orders of genome architecture. However, there are still lots of questions. What is the relationship between individual lncRNAs-induced alterations of 3D genome architecture and promiscuous RNA-mediated PRC2 recruitment? Individual lncRNAs-induced changes of 3D genome architecture are the causes or the results of alterations in promiscuous RNA-mediated PRC2 recruitment. Thus understanding the relationship between individual lncRNAs binding and promiscuous lncRNAs binding by PRC2 is essential to clarify the role of PRC2-meidated epigenetic landscape in heart development.

## 5 RNA with the G4 structure controls heart development

The RNA helicase RHAU resolves mRNA G4 structures. Cardiac deletion of *Rhau* leads to heart defects and embryonic lethality in mice (Nie et al., 2015). This suggested that RNA with the G4 structure is essential for heart development. LncRNA *Bvht* contains a G-rich motif which is essential for heart development and cardiovascular lineage decisions. The nucleic acid chaperone cellular nucleic acid binding protein that promotes the formation of G4 structures and is also essential for heart development (Xue et al., 2016). These results suggest that RNA with the G4 structure is essential for heart development.

There are 987 PRC2-interacted RNA transcripts with predicted G4 structures in mouse ESCs (Beltran et al., 2019). The dynamic expressions of these RNAs during heart development is still unknown. Thus it is still difficult to understand the expression pattern and functions of individual lncRNAs with G4 structure during heart development.

Telomeric repeat-containing RNA (TERRA) is an lncRNA transcribed from telomeres, which contains UUAGGG repeats as a G4 noncoding RNA (Takahama et al., 2013). The interaction between TERRA and PRC2 could regulate PRC2 locations in genome *via* G4 structure (Montero et al., 2018; Zhang et al., 2019, 2). Some reports suggest that TERRA plays a key role in regulating the transcriptional landscape of pluripotent cells by binding to PRC2 (Marión et al., 2019). The premature expression of heart development-associated genes has been observed in TERRA knockdown ESCs by RNA-seq (Chu et al., 2017). Thus, TERRA, a G4 structure-enriched lncRNA, may control genome-wide PRC2 occupancy in ESCs. However, there is still no direct evidence that TERRA functions in heart development and that TERRA changes the promiscuous RNA binding profile by PRC2 during heart development.

In summary, RNA with the G4 structure is essential for heart development and RNA with G4 structure may regulate the PRC2-mediated epigenetic landscape in ESCs and early embryonic development.

## 6 Discussion

The binding of individual lncRNAs and promiscuous RNA binding by PRC2 may both play key roles in controlling heart development. The relationship between binding of individual lncRNA and promiscuous RNA by PRC2 in heart development is therefore an interesting unknown question. In addition, whether individual lncRNAs regulate promiscuous RNA binding by PRC2 during heart development is still unknown. How do binding of individual lncRNA and promiscuous RNA by PRC2 coordinately construct the dynamic epigenetic landscape?

**FIGURE 4:**
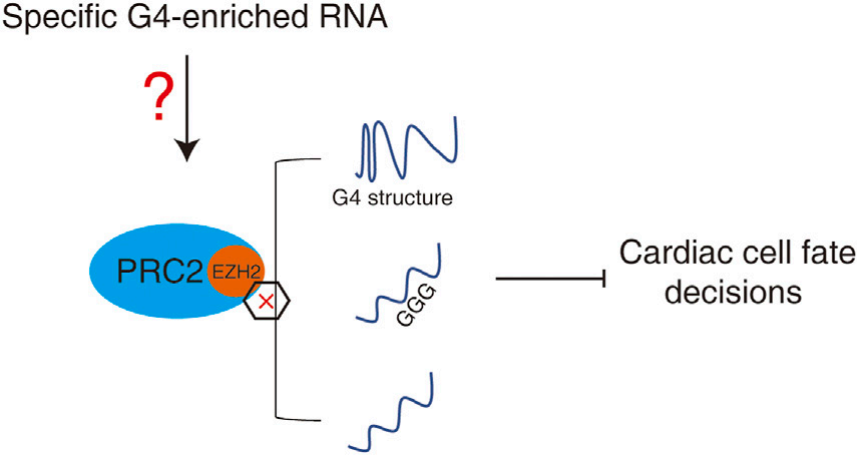
A “competitive model” to regulate promiscuous RNA by PRC2 during heart development or early embryonic development. Dynamic expression of specific G4-enriched RNAs may competitively control the interaction between PRC2 and other RNAs, and therefore control the promiscuous RNAs-mediated PRC2-dependent epigenetic landscape.

Since the binding priority of RNA to PRC2 is different, a “competitive model” may be described to explain the relationship between binding of individual lncRNA and promiscuous RNA by PRC2 during heart development and early embryonic development (Figure 4). During heart development, the dynamic expression of specific G4-enriched lncRNAs may competitively control the interaction between PRC2 and other RNAs. The dynamic changes in one or a few specific G4-enriched lncRNAs may thus control the promiscuous RNA binding profile by PRC2 which further regulates the PRC2-meidated epigenetic landscape during heart development.

## 7 Conclusion

PRC2- and H3K27me3-mediated epigenetic landscape regulate cardiac cell fate decisions. Both individual and promiscuous RNA binding by PRC2 is essential for cardiac cell fate decisions. RNA with the G4 structure is also important for heart development. The binding affinity between PRC2 and RNA is not dependent on a specific protein-binding motif in RNA but rather depend on the amount of guanine nucleotides and specific G4 secondary RNA structure. Thus the machinery by which promiscuous RNA binding by PRC2 controls the well-ordered cardiac cell lineage differentiation process becomes an interesting open question. A “competitive model” among PRC2-interatcting individual RNAs may explain the coordinate regulation of the well-ordered cardiac cell lineage differentiation process. Dynamic specific G4-enriched lncRNAs may act during heart development to change the promiscuous RNA binding by PRC2.

Thus, systemic understanding of stage-specific promiscuous RNA binding profiles, the dynamic expression of specific G4-enriched lncRNAs, and specific G4-enriched lncRNA-induced changes in the RNA binding profile by PRC2 during heart development will be important for comprehension of the dynamic epigenetic landscape regulating heart development. Finally, this will be essential for understanding the pathogenesis of genetic and non-genetic factors-induced congenital heart disease.
